# Nitrate and a nitrate-reducing *Rothia aeria* strain as potential prebiotic or synbiotic treatments for periodontitis

**DOI:** 10.1038/s41522-023-00406-3

**Published:** 2023-06-17

**Authors:** Danuta Mazurel, Miguel Carda-Diéguez, Thomas Langenburg, Miglė Žiemytė, William Johnston, Carlos Palazón Martínez, Fernando Albalat, Carmen Llena, Nezar Al-Hebshi, Shauna Culshaw, Alex Mira, Bob T. Rosier

**Affiliations:** 1 Genomics & Health Department, FISABIO Institute, Valencia, Spain; 2 Department of Preventive Dentistry, Academic Center for Dentistry Amsterdam (ACTA), University of Amsterdam and VU University Amsterdam, Amsterdam, Netherlands; 3 Department of Biological and Biomedical Sciences, Glasgow Caledonian University, Glasgow, UK; 4 Centro Periodontal de Valencia, Valencia, Spain; 5 Department of Stomatology, University of Valencia, Valencia, Spain; 6 Oral Microbiome Research Laboratory, Kornberg School of Dentistry, Temple University, Philadelphia, PA USA; 7 Oral Sciences, Glasgow Dental Hospital and School, School of Medicine, Dentistry and Nursing, College of Medical, Veterinary and Life Sciences, University of Glasgow, Glasgow, UK

**Keywords:** Clinical microbiology, Microbiome, Dentistry, Microbial ecology

## Abstract

A few studies indicate that nitrate can reduce dysbiosis from a periodontitis point of view. However, these experiments were performed on samples from healthy individuals, and it is unknown if nitrate will be effective in periodontal patients, where the presence of nitrate-reducing bacteria is clearly reduced. The aim of this study was to test the effect of nitrate and a nitrate-reducing *R. aeria* (Ra9) on subgingival biofilms of patients with periodontitis. For this, subgingival plaque was incubated with 5 mM nitrate for 7 h (*n* = 20) or 50 mM nitrate for 12 h (*n* = 10), achieving a ~50% of nitrate reduction in each case. Additionally, Ra9 was combined with 5 mM nitrate (*n* = 11), increasing the nitrate reduced and nitrite produced (both *p* < 0.05). The addition of nitrate to periodontitis communities decreased biofilm mass (50 mM > 5 mM, both *p* < 0.05). Five millimolar nitrate, 50 mM nitrate and 5 mM nitrate + Ra9 led to 3, 28 and 20 significant changes in species abundance, respectively, which were mostly decreases in periodontitis-associated species. These changes led to a respective 15%, 63% (both *p* < 0.05) and 6% (not significant) decrease in the dysbiosis index. Using a 10-species biofilm model, decreases in periodontitis-associated species in the presence of nitrate were confirmed by qPCR (all *p* < 0.05). In conclusion, nitrate metabolism can reduce dysbiosis and biofilm growth of periodontitis communities. Five millimolar nitrate (which can be found in saliva after vegetable intake) was sufficient, while increasing this concentration to 50 mM (which could be achieved by topical applications such as a periodontal gel) increased the positive effects. Ra9 increased the nitrate metabolism of periodontitis communities and should be tested in vivo.

## Introduction

In susceptible individuals, periodontal diseases can develop as a result of an inflammatory response to dental plaque accumulation^1^. As the biofilm becomes thicker and inflammation increases, environmental changes can stimulate dysbiosis by selecting for anaerobic, inflammation-tolerant, proteolytic and/or alkaliphilic species^2^. The initial stage of periodontal disease is gingivitis, which is a mostly reversible inflammation of the gums present in most adolescents^3^. Long-lasting or repeated episodes of gingivitis can lead to periodontitis, which is a chronic and destructive inflammatory disease. Periodontitis may affect up to 50% of the adult population, with ~10% suffering from severe periodontitis^4^.

Within the oral cavity, periodontitis can be painful in some cases, cause halitosis and ultimately lead to tooth loss. In addition, emerging evidence suggests that periodontitis’ consequences extend beyond the oral cavity. For example, periodontitis is associated with an increased risk of diabetes^5^, rheumatoid arthritis^6^, atherosclerosis^7^, and hypertension^8^. Current periodontitis treatments are resource-intensive, time-consuming, and often only partially successful^9^. Inflammation and dysbiosis are reduced after periodontal treatment, but frequently reappear over the following months. Thus, new strategies to improve treatment efficiency are required to reduce the global health and financial burdens of periodontitis. In this respect, novel treatments such as prebiotics and probiotics that reduce dysbiosis (or stimulate eubiosis) and decrease inflammation should be explored^2,9^.

Periodontitis-associated microbiota composition is diverse and includes different species of the phyla Bacteroidota (formerly known as Bacteroidetes), Candidatus Saccharibacteria (formerly Candidate Division TM7), Bacillota (formerly Firmicutes), Pseudomonadota (formerly Proteobacteria), Spirochaetes, and Synergistetes^10^. Some of the species with the strongest association with periodontitis include the periodontal “red complex” (*Porphyromonas gingivalis*, *Treponema denticola*, and *Tannerella forsythia*) in which different potential virulence mechanisms have been identified^11^. In 2014, Pérez-Chaparro et al.^10^ systematically reviewed the literature, identifying 17 species associated with periodontitis. In addition to an increase in disease-associated species, health-associated species decrease in periodontitis, and their proportion is recovered after periodontal treatment in most patients^9^. Recently, Chen et al. developed the subgingival microbial dysbiosis index (SMDI) (i.e., a machine learning-based index assessing periodontal health in the subgingival plaque bacteriome) that calculates the amount of dysbiosis based on the abundance of different disease- and health-associated species^12^. Interestingly, these authors found that nitrate has dysbiosis-lowering properties when applying their index to in vitro data from Rosier et al.^13^ and a clinical study by Jockel-Schneider et al.^14^. In the latter clinical study, nitrate intake by patients with chronic gingivitis also reduced gingival inflammation^15^.

Several potential mechanisms of nitrate have been described that could improve periodontal health (reviewed by Rosier et al.^16^). In short, oral bacteria reduce nitrate to nitrite and nitric oxide—a host signalling molecule with antimicrobial activity. On the one hand, nitric oxide could directly reduce inflammatory pathways of host cells. In addition, nitric oxide can kill periodontitis-associated species (e.g., *Porphyromonas gingivalis* and *Aggregatibacter actinomycetemcomitans* have been shown to be sensitive to nitric oxide)^17^. In contrast, periodontal health-associated *Rothia* and *Neisseria* have been found to correlate negatively with inflammatory cytokines^18–20^ and their increase in the presence of nitrate could reduce inflammation. Importantly, current studies focusing on nitrate and oral health have only included patients without periodontitis. It is therefore important to determine how nitrate affects the bacterial composition in periodontitis. Additionally, as it is known that nitrate-reducing bacteria (e.g., *Rothia dentocariosa* and *Rothia aeria*) decrease in periodontitis and can increase after periodontal treatment^9,12,21^, it should be tested if nitrate-reducing probiotics have additional benefits.

The aim of this study was therefore to test the effect of nitrate and a nitrate-reducing *Rothia aeria* CECT9999 (Ra9) strain on periodontitis communities in vitro. For this, fresh subgingival plaque samples of periodontitis patients were grown ex vivo in an impedance based system that measures real-time biofilm growth^22^. Subgingival biofilm growth was monitored under a physiologically relevant nitrate concentration of 5 mM in the presence or absence of the Ra9 strain, or a high nitrate concentration of 50 mM, which could be obtained by the topical application of nitrate. Supernatant samples were taken for the measurements of nitrate, nitrite and pH. The remaining biofilms were collected for DNA isolation and Illumina sequencing of the 16S rRNA gene. Finally, the effect of nitrate on periodontitis-associated species was further studied by monitoring changes in a 10-species biofilm model using qPCR.

## Results

### Effect of nitrate with or without probiotic candidate on periodontal biofilm growth

**Fig. 1:**
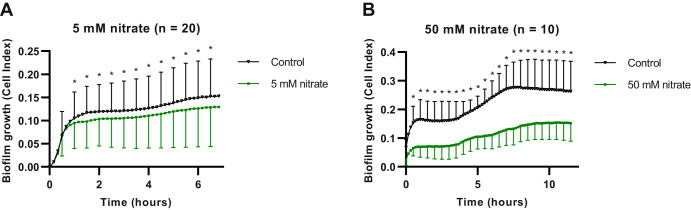
Effect of nitrate on periodontal biofilm formation. Real-time 7 or 12 h growth curves of in vitro periodontal plaque biofilms under different treatment conditions, namely (**A**) samples supplemented with or without 5 mM nitrate (*n* = 20), or (**B**) with or without 50 mM nitrate (*n* = 10). Biofilm formation is expressed as Cell Index (CI) as determined using impedance measurements. Standard deviations are shown at 30 min intervals. The error bars represent the standard deviation of the mean. Statistically significant differences (*p*-value < 0.05 according to Wilcoxon matched pairs signed-rank test) are marked by an asterisk (*). The addition of the Ra9 strain appeared to affect initial biofilm formation, but had no significant effect on the final biofilm quantity (Supplementary Fig. 1).

**Fig. 2:**
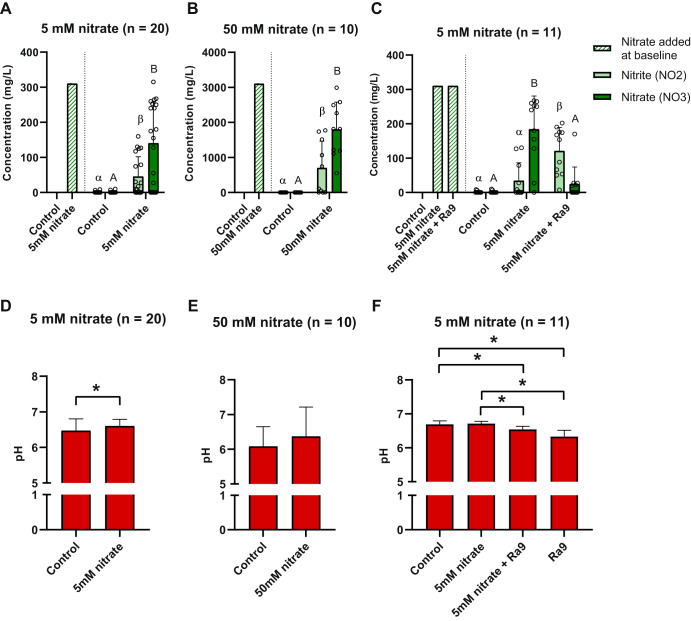
Effect of nitrate and *Rothia aeria* supplementation on in vitro periodontal community metabolism. Concentration of nitrate and nitrite in the supernatant of (**A**) biofilms supplemented with or without 5 mM nitrate (*n* = 20), after 7 h of biofilm growth, (**B**) with or without 50 mM nitrate (*n* = 10), after 12 h of biofilm growth, or (**C**) biofilms supplemented with or without 5 mM nitrate or 5 mM nitrate plus Ra9 (*n* = 11), after 7 h of biofilm growth. Supernatant pH of (**D**) biofilms supplemented with or without 5 mM nitrate, after 7 h of biofilm growth (*n* = 20), and (**E**) with or without 50 mM nitrate (*n* = 10), after 12 h of biofilm growth, or (**F**) biofilms (*n* = 11) supplemented with or without 5 mM nitrate or 5 mM nitrate plus Ra9, after 7 h of biofilm growth. In Fig. 2A–C the striped bar on the left side of the graph illustrates the concentration of nitrate added at baseline. Circles represent individual samples. The error bars represent the standard deviation of the mean. An asterisk (*) or distinct letters are used to indicate a statistically significant difference between treatment conditions in supernatant nitrite and pH (uppercase letters) or supernatant nitrate (Greek letters) (*p*-value < 0.05 according to Wilcoxon matched-pairs signed rank test, with Bonferroni correction for multiple comparisons for Fig. 2C, F). Ra9: *Rothia aeria* CECT9999.

Subgingival plaque samples of periodontitis patients (patient information can be found in Supplementary Table 1) were incubated with nitrate. The incubation time for experiments with 5 mM (*n* = 20) and 50 mM nitrate (*n* = 10) was 7 h and 12 h (Table 1, Fig. 1), respectively. These timepoints were selected to reach a similar percentage (~50%) of nitrate reduction for each concentration of nitrate (Fig. 2A, B). Biofilm attachment and growth of in vitro periodontal plaque samples were monitored in real-time by impedance measurements under different growth conditions (Table 1). Figure 1 shows the average growth curve of the biofilms over time, expressed as Cell Index (CI) values^22^. Biofilm growth of communities grown in the presence of 50 mM nitrate was significantly lower than in the paired control samples at all timepoints after zero (all *p* < 0.05). In the case of the biofilms grown with 5 mM nitrate, a significant decrease in biofilm growth was observed from 1 h until the last timepoint (7 h, *p* < 0.05), indicating that the quantity of the final biofilms was lower.

**Table 1:** Overview of experimental parameters of in vitro periodontal plaque biofilm growth in the impedance system

Treatment group	Donors	Growth time	Treatment conditions
5 mM nitrate (*n* = 20)	*n* = 9	7 h	Control and 5 mM nitrate
	*n* = 11	7 h	Control, 5 mM nitrate, 5 mM nitrate + Ra9, Ra9
50 mM nitrate (*n* = 10)	*n* = 10	12 h	Control and 50 mM nitrate

Ra9: *Rothia aeria* CECT9999.

Apart from the 5 mM and 50 mM nitrate conditions, the effect of a nitrate-reducing probiotic in the presence or absence of 5 mM nitrate was determined (*n* = 11). The addition of the probiotic candidate Ra9 with or without nitrate appeared to affect initial biofilm formation, but did not significantly affect the final biofilm quantity compared to the untreated control (Supplementary Fig. 1). When comparing the probiotic condition with and without 5 mM nitrate, no significant difference was observed in biofilm growth.

### Effect of nitrate and probiotic candidate addition on denitrification and pH

To determine the nitrate reduction capacity (NRC) of the in vitro communities, nitrate and nitrite concentrations were determined in the supernatant of all samples collected after in vitro biofilm growth. Figure 2A–C show that nitrate, when supplied to the medium at a concentration of 5 mM (310 mg/L) or 50 mM (3100 mg/L), was partly reduced at the end of the biofilm growth period (7 h and 12 h respectively). These timepoints were selected to reach a similar percentage of nitrate reduction, leading to 56% reduction of the 5 mM nitrate (Fig. 2A) and 42% of the 50 mM nitrate (Fig. 2B) after 7 h and 12 h, respectively. The addition of the nitrate-reducing probiotic candidate significantly enhanced the nitrate reduction capacity of the periodontitis communities (Fig. 2C). Biofilms supplemented with 5 mM nitrate plus the probiotic reduced 97% of nitrate added after 7 h (*p* < 0.001 compared to the biofilms supplemented with 5 mM nitrate alone).

The reduction of nitrate to nitrite occurs at an equimolar ratio, i.e., a production of 5 mM (220 mg/L) nitrite from 5 mM (310 mg/L) nitrate, after which nitrite can be further reduced to form, for example, nitric oxide or ammonium^16^. Therefore, in parallel to the reduction in nitrate, an increase in the concentration of nitrite was observed in the 5 mM and 50 mM nitrate treatment conditions (both *p* < 0.05). After 7 h, 34% (i.e., ~1.7 mM) of nitrite produced had been further reduced in samples treated with 5 mM nitrate (Fig. 2A). In samples treated with 50 mM nitrate after 12 h, 10% (i.e., ~5 mM) of the nitrite produced had been further reduced (Fig. 2B). The increased nitrate reduction in the presence of the probiotic also increased the concentration of nitrite detected after 7 h compared to samples treated with 5 mM nitrate only (Fig. 2C).

A concentration of 5 mM nitrate caused a small but significant increase in pH (pH 6.6 ± 0.19 compared with 6.5 ± 0.33 of the control condition, *p* < 0.05), while no significant effect of 50 mM nitrate was observed on supernatant pH at the end of the biofilm growth period (Fig. 2D, E). The addition of the Ra9 strain in combination with 5 mM nitrate or the Ra9 strain alone resulted in a small but significant decrease in pH (pH 6.5 ± 0.09 and 6.3 ± 0.18 respectively, compared with pH 6.7 ± 0.10 of the control condition, both *p* < 0.01) (Fig. 2F).

### The effect of nitrate and probiotic candidate addition on bacterial composition

**Fig. 3:**
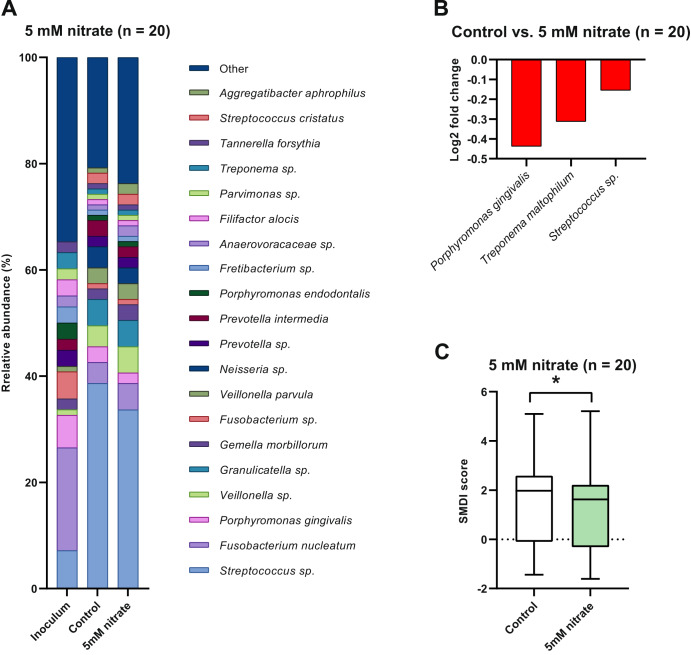
Effect of 5 mM nitrate supplementation on in vitro periodontal biofilm composition. Bacterial composition of periodontal plaque biofilms grown in vitro supplemented with or without 5 mM nitrate (*n* = 20). **A** Relative abundance at the species taxonomic level as determined by 16 S rRNA gene sequencing. The top 20 most abundant species are shown and sorted by overall mean abundance; all other species are included as “other”. **B** Log2 fold-change of relative abundance of all species with statistically significant changes (ANCOM-BC adjusted *p*-value < 0.05). Species identified by the Boruta method as a significantly relevant feature are marked (#). **C** Box and whisker plot of the dysbiosis index. The dysbiosis index is expressed as SMDI score^12^. Plotted are the median, 25th to 75th percentile (box) and lowest to highest value (whiskers). A statistically significant difference (*p*-value < 0.05 according to Wilcoxon matched-pairs signed rank test) is marked by an asterisk (*).

**Fig. 4:**
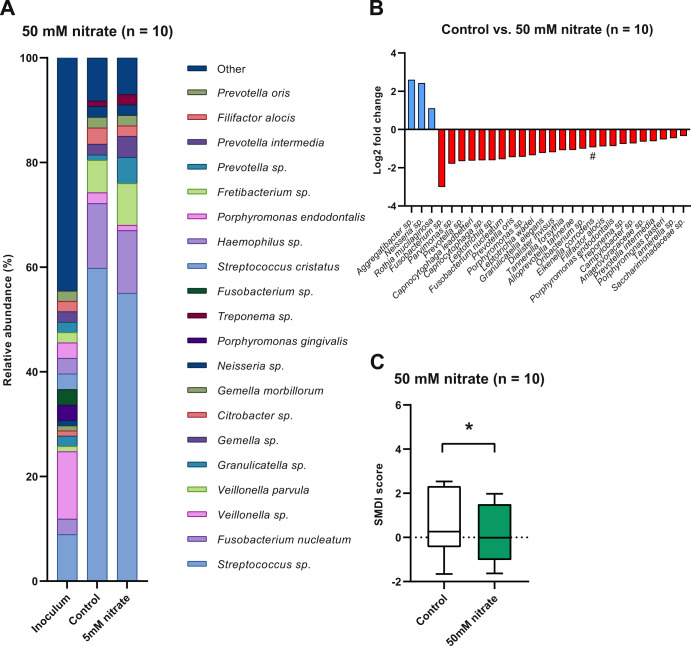
Effect of 50 mM nitrate supplementation on in vitro periodontal biofilm composition. Bacterial composition of periodontal plaque biofilms grown in vitro supplemented with or without 50 mM nitrate (*n* = 10). **A** Relative abundance at the species taxonomic level as determined by 16 S rRNA gene sequencing. The top 20 most abundant species are shown and sorted by overall mean abundance; all other species are included as “other”. **B** Log2 fold-change of relative abundance of all species with significant changes (ANCOM-BC adjusted *p*-value < 0.05). Species identified by the Boruta method as a significantly relevant feature are marked (#). **C** Box and whisker plot of the dysbiosis index. The dysbiosis index is expressed as SMDI score^12^. Plotted are the median, 25th to 75th percentile (box) and lowest to highest value (whiskers). A statistically significant difference (*p*-value < 0.05 according to Wilcoxon matched-pairs signed rank test) is marked by an asterisk (*).

The effect of nitrate and the Ra9 strain on the bacterial composition of the in vitro periodontal plaque biofilms was assessed by 16S rRNA gene sequencing analysis. Both in the control as well as in the 5 mM nitrate-treated biofilms a *Streptococcus* sp., *Fusobacterium nucleatum*, *Porphyromonas gingivalis* and a *Veillonella* sp. were the most dominant taxa (Fig. 3A). Similar species were found at high abundance in samples treated with 50 mM nitrate (a *Streptococcus* sp., *Fusobacterium nucleatum*, a *Veillonella* sp., and *Veillonella parvula*) (Fig. 4A). The bacterial composition of the periodontal biofilm samples reflects the clinical origin, as known periodontitis-related bacteria such as *Porphyromonas gingivalis*, *Treponema denticola*, *Tannerella forsythia* (“red complex” bacteria) and/or other species of these genera were also well represented in the biofilms.

Comparison of the general microbial composition (Supplementary Fig. 2) of biofilms receiving different treatments indicates no significant effect of the addition of nitrate or nitrate plus the probiotic candidate Ra9 on biofilm richness and evenness as expressed by Shannon diversity indexes (Supplementary Fig. 2D–F). Furthermore, CCA analysis showed no significant separation between control and nitrate treatment conditions (Supplementary Fig. 2A, B). For samples treated with Ra9 with or without nitrate, a significant separation between microbial communities is observed in the CCA plot (Supplementary Fig. 2C).

**Fig. 5:**
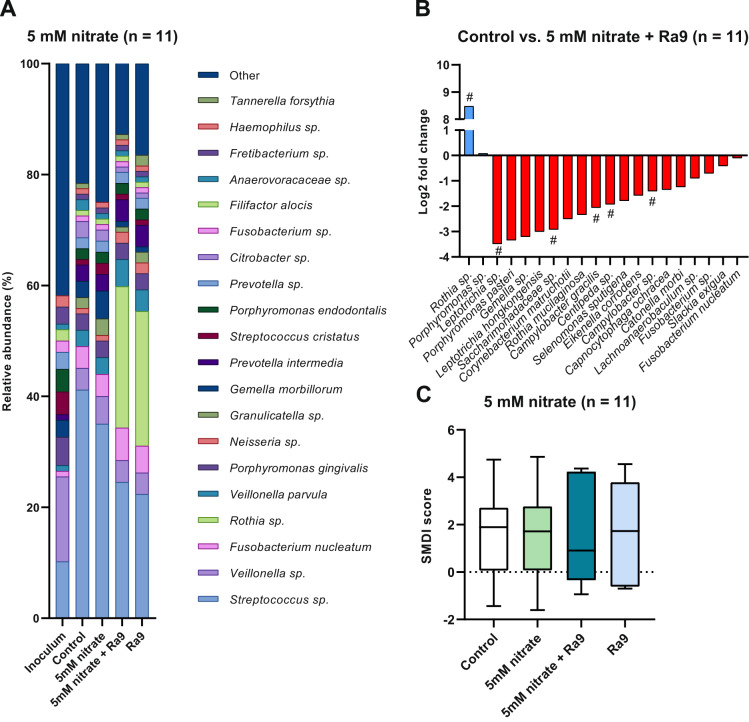
Effect of nitrate and *Rothia aeria* supplementation on in vitro periodontal biofilm composition. Bacterial composition of periodontal plaque biofilms grown in vitro supplemented with or without 5 mM nitrate and Ra9 (*n* = 11). **A** Relative abundance at species taxonomic level as determined by 16 S rRNA gene sequencing. The top 20 most abundant species are shown and sorted by overall mean abundance; all other species are included as “other”. **B** Log2 fold-change of relative abundance of all species showing significant changes (ANCOM-BC adjusted *p*-value < 0.05). Species identified by the Boruta method as a significantly relevant feature are marked (#). **C** Box and whisker plot of the dysbiosis index. The dysbiosis index is expressed as SMDI score^12^. Plotted are the median, 25th to 75th percentile (box) and lowest to highest value (whiskers). Differences are not statistically significant (adjusted p-value > 0.05 according to Wilcoxon matched-pairs signed rank test). Ra9: *Rothia aeria* CECT9999.

At the level of individual species, significant changes (all *p* < 0.05) can be observed between treatment conditions, shown as Log2 fold-changes in Figs. 3B, 4B and 5B. In general, disease-associated species appear to decrease when adding nitrate (50 mM > 5 mM) or nitrate combined with the Ra9 strain. Treatment using 5 mM nitrate (Fig. 3B) resulted in a decreased abundance of *Porphyromonas gingivalis*, *Treponema maltophilum*, and an unassigned *Streptococcus* sp., compared to controls. The 10-fold higher 50 mM nitrate treatment (Fig. 4B) increased the relative abundance of a *Rothia* sp. as well as an *Aggregatibacter* sp. (other than the known disease-associated species *Aggregatibacter actinomycetemcomitans*, which is detected separately and does not change significantly) and a *Neisseria* sp., whereas 25 species decreased in abundance, including multiple species of *Fusobacterium*, *Porphyromonas*, and *Prevotella*, among others (all *p* < 0.05). Specifically, in samples treated with 50 mM nitrate, the classified species that decreased significantly were *Capnocytophaga leadbetteri*, *Fusobacterium nucleatum*, *Prevotella oris*, *Leptotrichia wadei*, *Granulicatella elegans*, *Dialister invisus*, *Tannerella forsythia*, *Alloprevotella tannerae*, *Eikenella corrodens*, *Filifactor alocis*, *Porphyromonas endodontalis*, *Prevotella intermedia* and *Porphyromonas pasteri*. The addition of the probiotic candidate Ra9 in samples treated with 5 mM nitrate (Fig. 5B) also resulted in a significant increase of a *Rothia* sp. (though not classified, this is likely to correspond to *Rothia aeria*, resulting from the added Ra9 strain). *Rothia mucilaginos*a, a species that increased in samples treated with 50 mM nitrate, decreased in samples treated with both 5 mM nitrate and the probiotic (*p* < 0.05), possibly resulting from nutrient and niche competition with *Rothia aeria*. In addition, the abundance of 17 species decreased (all *p* < 0.05), among which we find disease-associated species such as *Selenomonas sputigena*, *Slackia exigua* and *Fusobacterium nucleatum*.

### The effect of nitrate and probiotic candidate addition on the dysbiosis index

The dysbiosis level of the in vitro periodontal plaque biofilms was determined by computing the SMDI score^12^. Treatment of in vitro periodontal plaque biofilm with 5 mM nitrate or 50 mM nitrate significantly reduced the SMDI score compared to controls (Figs. 3C and 4C). This reduction was a 0.25 reduction (15%) in SMDI score for 5 mM nitrate (after 7 h) and a 0.35 reduction (63%) in SMDI score for 50 mM nitrate (after 12 h). Interestingly, when adding Ra9 in combination with 5 mM nitrate (synbiotic treatment), the SMDI only decreased 6% and this decrease was not significant, possibly resulting from Ra9 decreasing the abundance of health-associated species with a similar metabolism (e.g., *Rothia mucilaginosa*, which is one of the top health-associated species that affect the SMDI score^12^).

### The effect of nitrate on a 10-species biofilm model

**Fig. 6:**
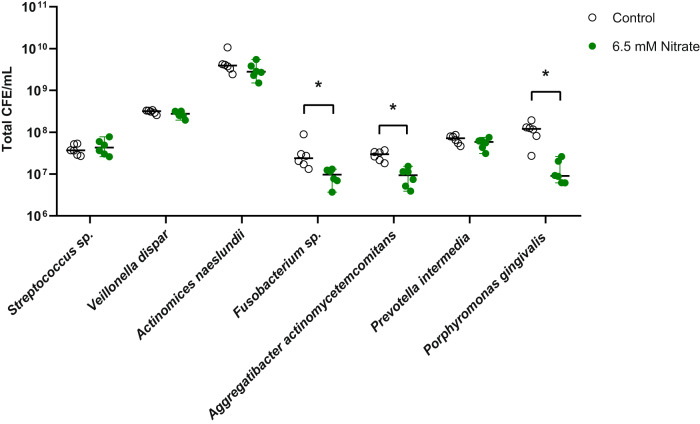
Changes in 10-species biofilm composition in the presence of nitrate. qPCR quantification of total colony forming equivalents (CFE) of bacterial species from a 10-species in vitro periodontal biofilm model^23^ treated with 6.5 mM nitrate compared to control biofilms. In this model, *Veillonella dispar* and *Actinomyces naeslundii* are nitrate-reducing species. Significant differences (adjusted *p*-value < 0.05 according to Mann–Whitney *U*-test with Bonferroni–Dunn multiple testing correction) are marked by an asterisk (*). The error bars represent the standard deviation of the mean.

The sequencing results of periodontal plaque grown with nitrate indicated that nitrate could decrease the quantity of anaerobic bacteria associated with periodontitis. To confirm this possibility, a multi-species in vitro biofilm model containing a synthetic community of 10 oral strains was used to study the effect of nitrate on the growth of specific bacterial species. qPCR quantification of this 10-species community suggests that nitrate significantly reduced the growth of specific periodontitis-associated bacteria, including *Fusobacterium nucleatum*, *Aggregatibacter actinomycetemcomitans* and *Porphyromonas gingivalis* (all *p* < 0.05, Fig. 6).

## Discussion

Evidence from the last decade indicates that nitrate metabolism can stimulate eubiosis in individuals without periodontitis^16^. However, this is the first study in which the effect of nitrate on dysbiotic communities of patients with periodontitis was explored. Both 5 mM nitrate (a physiologically relevant concentration) and 50 mM nitrate (a concentration that could be obtained by the topical application of nitrate) decreased the biofilm quantity and dysbiosis index of the biofilms, indicating a beneficial effect on both bacterial growth and composition. A lower amount of subgingival plaque with a more health-associated composition is likely to reduce inflammation in vivo. The 50 mM concentration of nitrate led to more significant decreases in periodontitis-associated species and an additional reduction of biofilm accumulation compared with 5 mM nitrate. We suggest that the effect of nitrate-rich vegetable intake, as well as the topical application of nitrate in periodontal pockets that leads to a local high nitrate concentration (e.g., by using a periodontal gel with nitrate and antioxidants), should thus be explored in vivo. When adding the *Rothia aeria* CECT9999 (Ra9) strain to the 5 mM nitrate condition, more nitrate was reduced, and more nitrite was produced, which was accompanied by more significant changes in biofilm composition. An increase in nitrite production could improve systemic health by increasing systemic nitric oxide levels, which can improve conditions such as hypertension and diabetes—both associated with periodontitis^5,8^. This in vitro study indicates that nitrate as a prebiotic and nitrate together with a nitrate-reducing probiotic (i.e., a synbiotic combination) are promising (adjunct) treatments for periodontitis.

In this study, subgingival plaque samples of 30 periodontitis patients were divided in three experiments: 5 mM nitrate (*n* = 9), 5 mM nitrate in combination with the Ra9 strain (*n* = 11) and 50 mM nitrate (*n* = 10). All experiments contained relevant control conditions, and in the second group of 11 patients, a 5 mM nitrate condition without the *Rothia aeria* strain was added, leading to a total of 20 individuals treated with only 5 mM nitrate (Table 1). Subgingival plaque incubated with 5 mM nitrate was grown for 7 h (the same time as used previously for healthy communities by Rosier et al.^24^) and communities incubated with 50 mM nitrate were grown 12 h, both growth periods allowing for around 50% reduction of the different nitrate concentrations.

The final biofilm mass of the communities grown for 7 h with 5 mM nitrate was significantly reduced by 15% (*p* < 0.05), while after 7 h of growth, the biofilms for the communities grown with 50 mM were reduced by 53% (quantified in real time) (*p* < 0.05). After 12 h, the final cell index of the biofilms grown with 50 mM nitrate remained 42% lower than the control condition (*p* < 0.05). Dental plaque accumulation is considered the main stress factor in periodontitis development^1,2,25^. The host responds to the accumulated biofilms with gingival inflammation that can change the environment and give a selective advantage to periodontitis-associated proteolytic species^1,2^. This study indicates that nitrate could decrease the accumulation of dysbiotic periodontal plaque, which was not observed previously when adding nitrate to healthy communities^13^. Nitrate could reduce biofilm growth of sensitive bacteria because of the antimicrobial properties of its reduction products, such as nitric oxide^17^, which is also a biofilm dispersal signal for different bacteria^2^. Oral bacteria have been shown to have different tolerance levels of nitrate, nitrite and nitric oxide^17,26^, potentially explaining variations in responses of healthy biofilms compared with dysbiotic communities. Thus far this has been the only study testing the effect of nitrate on periodontal community growth. To our knowledge, there is only one study where the effect of nitrate intake on plaque accumulation was tested in vivo: Jockel-Schneider et al. tested the effect of nitrate-rich lettuce juice intake (compared to a placebo) on different clinical parameters and observed a trend of decrease in plaque control record^15^. The current data thus suggest that the effect of nitrate on plaque accumulation in patients with periodontal diseases should be further explored.

Apart from a decrease in biofilm quantity, the microbial composition of the biofilm affects inflammation, as some species stimulate inflammatory responses whereas others reduce inflammation^27^. In our study, both 5 mM nitrate and 50 mM nitrate significantly reduced the dysbiosis index in the final biofilms by 15% and 63%, respectively. This is in accordance with a previous study that showed that the SMDI dysbiosis index of healthy communities decreased after 5 h of growth with 6.5 mM nitrate in vitro^12,13^. Additionally, the dysbiosis index decreased in patients with chronic gingivitis consuming lettuce juice regularly during 2 weeks^12,14^. In our study, the addition of 5 mM nitrate led to a significant decrease of *Porphyromonas gingivalis*, *Treponema maltophilum* (both strongly associated with periodontitis), and an unclassified *Streptococcus* sp. *Streptococcus* spp. are generally periodontal health-associated (with the exception of *S. constellatus*), but decrease in the presence of nitrate in healthy individuals, possibly because nitrate could prevent the overgrowth of aciduric representatives^16^. The 50 mM nitrate condition increased an unclassified *Aggregatibacter* species, an unclassified *Neisseria* species and *Rothia mucilaginosa*, while decreasing 25 species of which most were associated with periodontitis on a genus- or species-level. *Rothia* and *Neisseria* spp. are considered periodontal-health associated^21^, while *Aggregatibacter* is often associated with periodontitis, because of the strong association of *A. actinomycetemcomitans* with periodontitis in some populations^28^. However, this genus can also contain species that are not disease-associated. Regarding this, *A. actinomycetemcomitans* was detected as a classified species and did not change significantly in the presence of nitrate. The classified species that significantly decreased in the presence of 50 mM nitrate included *Fusobacterium nucleatum*, *Dialister invisus*, *Tannerella forsythia*, *Alloprevotella tannerae*, *Eikenella corrodens*, *Filifactor alocis*, *Porphyromonas endodontalis* and *Prevotella intermedia*, which are strongly associated with periodontitis^10,12,29^ and contributed to a reduced dysbiosis index.

The observation that 50 mM nitrate caused a larger decrease in dysbiosis index than 5 mM nitrate, indicates that the topical application of nitrate to achieve this concentration should be tested in future studies. It should be noted that 100 mM nitrate (without further reduction to nitrite and other compounds) can be toxic for oral species^26^ and it is unknown if the 50 mM nitrate used in our experiment directly inhibited certain species (i.e. exhibiting an antiseptic or toxic effect on specific species instead of prebiotic effect that shifts to community towards eubiosis). Regarding this, some species that decreased in the presence of 50 mM nitrate (e.g., *Capnocytophaga leadbetteri* and *Granulicatella elegans*) were not periodontitis-associated. Nitrite and nitric oxide are often toxic for bacteria at lower doses than nitrate (e.g., *S. mutans* was inhibited by 100 mM nitrate but by 0.5 mM nitrite)^26^. The reduction of nitrate to nitrite and further metabolization to nitric oxide in our in vitro conditions could also explain the decrease of different species in the 5 mM and 50 mM nitrate conditions. For example, the strongly periodontitis-associated *Porphyromonas gingivali*s can be killed by nitric oxide^17^ and decreased in both conditions.

The concentration of 5 mM nitrate can be obtained in saliva by vegetable consumption, which is considered a safe and recommendable strategy to increase the nitrate levels inside the oral cavity^16^. To obtain higher levels of nitrate, topical applications could be used such as toothpaste or, for the application in periodontal pockets, a periodontal gel. The safety of higher levels of nitrate and nitrate-reducing probiotics has been discussed previously in Rosier et al.^16^ and 2020b^24^, respectively. In short, doses far below the acceptable daily intake (3.7 mg nitrate/kg of body weight, which is 222 mg for an adult of 60 kg) could be added to oral hygiene products to obtain high concentrations of nitrate. For example, a concentration of 50 mM can be obtained by adding 3.1 mg of nitrate to a dose of 1 ml periodontal gel. Additionally, nitrate-rich vegetable extracts could be used containing natural antioxidants, or nitrate salts could be combined with antioxidants (e.g., vitamin C), to prevent potential N-nitroso compound formation. This could be especially relevant for supragingival biofilms in which the pH can reach low levels, stimulating N-nitroso compound formation^16^, while in periodontal pockets the pH tends to be neutral or slightly alkaline. The possibility of N-nitroso compound formation and the inhibition with antioxidants should be investigated in future studies.

The Ra9 strain in combination with 5 mM nitrate resulted in more significant changes (20 in total) on a species-level compared with just 5 mM nitrate (3 significant changes). These included periodontitis-associated species (e.g., *Selenomonas sputigena*, *Eikenella corrodens* and *Fusobacterium nucleatum*), but also health-associated and (potentially) nitrate-reducing species (e.g., *Corynebacterium matruchotii* and *Rothia mucilaginosa*). The decrease in a variety of species could have resulted from nitric oxide produced by Ra9, which contains nitrite-reduction genes^24^ and/or other potential antimicrobial mechanisms of this bacterium. Genes of *Rothia* species involved in the production of antimicrobial peptides, enterobactin (a metal chelating siderophore with antimicrobial activity) and valinomycin (an antimicrobial ionophore) have been identified^30^, and the possibility of such mechanisms affecting the oral microbiota should be explored. Additionally, Ra9 could have competed with nutrients and other compounds (e.g., nitrate and lactic acid as electron donor) with species with a similar type of metabolism, such as *Rothia mucilaginosa*. Both *Rothia mucilaginosa* and *Corynebacterium matruchotii* are among the health-associated species that affect the dysbiosis index. This could explain why the mean dysbiosis index of communities treated with Ra9 and 5 mM nitrate did not decrease significantly. It should be noted that a small effect on pH was observed (i.e., after the 7 h incubation period, the supernatant was pH 6.3 when adding Ra9 and pH 6.5 when adding Ra9 in combination with nitrate compared with pH 6.7 of the control condition, both *p* < 0.05), possibly resulting from the metabolism of the added Ra9 strain or from changes in the activity of other members of the bacterial community. In future studies, pH decreases introduced by probiotics (e.g., the currently used acidogenic *Lactobacillus* spp.^31^ that are likely to induce a larger pH drop) should be investigated as they could affect alkalophilic periodontitis-associated species or (in some cases) stimulate enamel demineralization when pH ~5.5 is reached. Our data indicates that the effect of Ra9 on bacterium composition and inflammation should be further explored in vivo as potential pro-, post- or synbiotic treatment. A limitation of our in vitro model is that there is no interaction with human host cells, which respond to changes in microbial composition and metabolites, including nitric oxide^32^. Based on a limited amount of clinical studies focusing on oral microbiota composition, inflammatory cytokines and/or clinical parameters, both nitrate^15^ and *Rothia* spp. are associated with reduced gingival inflammation^14,18,20,33^.

A consistent observation in our study was the decrease of *Porphyromonas gingivalis* or other *Porphyromonas* species in the presence of nitrate (with the exception of 1 unclassified *Porphyromonas* species that increased slightly when treating the communities with Ra9 and 5 mM nitrate). Additionally, *Fusobacterium nucleatum* and an unclassified *Fusobacterium* species decreased, which is consistent with *P. gingivalis* and *F. nucleatum* being sensitive to the oxidative stress of nitric oxide^16^. To test the effect of nitrate on these species, an established 10-species biofilm model was used, including these two species and two nitrate-reducing species (*Veillonella dispar* and *Actinomyces naeslundii*). In this model, the bacteria are grown for 1 week to form a biofilm and the species are quantified by qPCR. The addition of 6.5 mM nitrate led to a significant decrease in *Porphyromonas gingivalis*, *Fusobacterium nucleatum* and *Aggregatibacter actinomycetemcomitans*. These three species are strongly associated with periodontitis, while two of them (*P. gingivalis* and *F. nucleatum*) are also associated with halitosis, and their decrease is undoubtedly a beneficial change for oral health. This further supports the idea that the unclassified *Porphyromonas* and *Aggregatibacter* species that increased in different nitrate conditions are not related to these periopathogens. In line with our results, in a previous study using saliva of healthy individuals, 6.5 mM nitrate also led to a decrease of *Porphyromonas* and *Fusobacterium* on a genus level. However, these results have not been confirmed in clinical studies in which nitrate intake increased *Rothia* and/or *Neisseria*, while it decreased *Streptococcus*, *Veillonella* and/or *Prevotella*^34,35^ (reviewed by Rosier et al.^16^). Larger clinical studies in which changes are monitored on a species- and strain-level are needed to conclude the effects of nitrate on the oral microbiota in individuals with and without oral diseases.

When adding Ra9 in combination with 5 mM nitrate, more nitrate was reduced, and more nitrite was produced. This could be very relevant for human health, as nitrite production could improve systemic conditions such as hypertension, metabolic syndrome and diabetes, and can increase endothelial function and sport performance^36^. Interestingly, periodontitis, which is unequivocally linked to lower levels of nitrate-reducing organisms in subgingival plaque, has been associated with an increased risk of these systemic conditions^37^. Additionally, preeclampsia is one of the conditions associated with periodontitis and a recent study indicated that this condition was associated with a decrease in oral nitrate-reducing bacteria^38^. Therefore, the relationship between periodontitis, nitrate reduction and systemic complications and diseases should be investigated in future studies, as lower levels of nitrate-reducing bacteria in periodontitis patients could lead to a decrease in systemic nitric oxide levels, increasing the risk of different systemic conditions. We propose that nitrate-reducing probiotics could therefore increase nitric oxide availability, thereby reducing the risk of systemic conditions and complications that are stimulated by a nitric oxide deficit. Additionally, it should be explored if nitrate-reducing probiotics could improve sport performance. Interestingly, nitrate has clear anti-caries effects^39^, while athletes have an increased caries prevalence due to high carbohydrate consumption^40^. Nitrate with or without nitrate-reducing bacteria as a prebiotic or synbiotic treatments should be further explored in athletes to improve sport performance and oral health.

In conclusion, our data indicates that nitrate and nitrate-reducing bacteria should be explored as pre-, pro- and synbiotic treatments for periodontitis and the systemic complications associated with this oral disease. We found that nitrate can reduce biofilm growth and periodontitis-associated species in dysbiotic communities from periodontal pockets.

## Methods

### Bacterial strains and growth conditions

*Rothia aeria* CECT9999 (Ra9, isolate D1P7 described in^24^), was used as a nitrate-reducing probiotic candidate. Prior to the experiment, Brain Heart Infusion (BHI) (Biolife, Deerfield, Illinois, USA) agar plates were inoculated with the strain, incubated for 48 h at 37 °C, and stored at 4 °C for up to 14 days. A single colony was transferred from the BHI agar plates to liquid BHI and incubated for 24–48 h at 37 °C before inoculation to the biofilm culture.

### Patient inclusion and sampling procedure

Bacterial samples from periodontal pockets were collected at the Lluis Alcanyis Foundation dental clinic of the University of Valencia (Valencia, Spain) and the private clinic Centro Periodontal de Valencia (Valencia, Spain). The study protocol was approved by the Ethics Committee of the University of Valencia (Spain) (H1547805836517) and all participants signed an informed consent prior to sample donation.

Thirty-one periodontitis patients were recruited at the clinics before starting treatment. The included patients were ≥18 years of age and had a sufficient number of teeth in each quadrant (≥5). An overview of patient characteristics is included in Supplemental Table 1. The diagnosis of periodontitis was determined in accordance with the guidelines of The American Academy of Periodontology; (AAP)^41^. Exclusion criteria were (i) the use of systemic antibiotics or antiseptic mouthwash in the last month, (ii) active systemic infection, (iii) periodontal treatment in the previous 6 months, (iv) and pregnancy. The patients included were 16 males and 14 females, leading to a total of 30 individuals of which 11 were smokers. The patients’ age ranged from 20–76, with a mean age of 49 ± 14. Their average deepest pocket depth was 6.4 ± 1.9 mm.

Subgingival plaque was collected from the periodontal pocket at five different periodontitis sites in each patient using 6–10 sterile paper points. Paper points were transferred to a 2 mL tube containing BHI medium (for usage within 1 h) or reduced transport medium^42^ (for usage within 1–12 h) and stored at ~4 °C in a styrofoam box for transportation. The samples were transported to the laboratory and tested within 1–12 h.

### Real-time in vitro biofilm growth of periodontal plaque

Real-time in vitro biofilm growth of oral microorganisms was assessed using the xCELLigence RTCA single plate system (ACEA Biosciences)^13,43^. When grown in this system, bacteria from fresh subgingival samples attach to the wells interfering with the electrical current of the electrodes, and the resulting impedance allows the real-time quantification of biofilm growth^22^. The amount of biofilm growth is measured and expressed as “Cell Index” values, which correlate with total biofilm mass^44^.

Baseline cell-sensor impedance measurements were performed with 100 µl liquid Brain Heart Infusion broth (BHI) medium (Biolife) supplemented with 2 mL/L liquid vitamin K1 (V3501), 5 mg/L hemin and 1 mg/L menadione (all Sigma-Aldrich, St. Louis, Missouri, USA). Three baseline measurements of the control medium were performed in a 96-well plate with an integrated microelectronic cell sensor array (E-plate 96, ACEA Biosciences, San Diego, California, USA) at 3-min intervals. After this, another 50 µl BHI medium was added, which was supplemented to obtain the following treatment groups: 5 mM nitrate (Thermo Fisher Scientific), 50 mM nitrate, 5 mM nitrate combined with the Ra9 strain (OD_600nm_ Ra9 in well = 0.075), or the Ra9 strain alone (OD_600nm_ Ra9 in well = 0.075) (see Table 1 for details). Finally, 100 µl of BHI medium containing periodontal plaque as inoculum was added from an individual bacterial inoculum sample per patient. This bacterial inoculum was prepared by vortexing the paper points for 1 min, discarding the paper points, centrifuging the periodontal plaque sample (1 min, 9660 xg), discarding the (transport) medium supernatant, and re-suspending the bacterial pellet in BHI medium (100 µl per paper point, which is 600–1000 µl for 6–10 paper points). Replicates of each condition were prepared for every patient.

Concisely, the subgingival plaque samples were grown in BHI with or without 5 mM nitrate medium (*n* = 9), 50 mM nitrate medium (*n* = 10), or 5 mM nitrate, 5 mM nitrate + Ra9, and Ra9 medium (*n* = 11) (Table 1). All plates were sealed with adhesive aluminium foil and incubated at 37 °C. Cell impedance measurements were performed at 10-min intervals. The total growth period was 7 h when using 5 mM nitrate (the same time as used previously for healthy communities by Rosier et al.,^24^) or 12 h for 50 mM nitrate to obtain a ~50% of nitrate reduction of each concentration. Supernatants, as well as the corresponding biofilms collected through resuspension using PBS, were collected from the 96-well plate after the final cell-sensor impedance measurement. All samples were stored at −20 °C.

### Determination of pH, nitrate, and nitrite concentrations

Supernatant collected from the in vitro biofilms was used to determine the pH levels and the concentration of nitrate and nitrite using the RQflex® 10 Reflectoquant® reflectometer (Merck KGaA), following Rosier et al.^13^. Manufacturer protocols were adjusted for micro-volume sample sizes, and verified using known concentrations. The supernatant was applied undiluted to pH test strips (reference no. 1169960001) or as a ≥ 10-fold dilution of supernatant in demineralized water to the nitrate (reference no. 1169710001) and the nitrite (reference no. 1169730001) test strips. Fifteen μl of the sample was directly pipetted onto each of the two reaction zones of a test strip, excess liquid was removed by tipping the side of the strip on a tissue, and the strip was incubated according to the manufacturer’s instructions.

### DNA isolation

Bacterial DNA was extracted for Illumina sequencing of the 16 S rRNA gene. Bacterial pellets (two replicates of biofilms after in vitro growth or the pellet of 100 μl inoculum) were resuspended in 100 μl PBS and sonicated in a RAYPA ultrasonic bath for 30 s. To lyse the samples, they were incubated with lysozyme (20 mg/ml; Thermomixer comfort, Eppendorf), lysostaphin (2000 units/mg protein; Sigma-Aldrich) and mutanolysin (4000 units/mg protein; Sigma-Aldrich) for 60 min at 37 °C^13^. Next, DNA was extracted by the MagNA Pure LC 2.0 Instrument (Roche Diagnostics, Barcelona, Spain) with the MagNA Pure LC DNA Isolation Kit III for Bacteria and Fungi (Roche Diagnostics GmbH) using standard manufacturer protocol. DNA was eluted in 100 μl elution buffer and, if necessary (e.g., for some inoculum samples), further concentrated with a Vivacon® 500 100.000 MWCO Hydrosart filter (Sartorius). A QubitTM 3 Fluorometer (ThermoScientific) was used to determine DNA concentration and samples were stored at −20 °C until used for sequencing.

### Illumina 16 S rRNA gene sequencing and analysis

DNA from biofilms and inocula were sequenced to determine the bacterial composition. Library preparation was performed according to the 16 S rDNA gene Metagenomic Sequencing Library Preparation Illumina protocol (Part #15044223 Rev. A, Illumina Inc.), using gene-specific 16 S amplicon PCR primers for the V3 and V4 region, resulting in amplicons of ~460 bp (F: 5' TCGTCGGCAGCGTCAGATGTGTATAAGAGACAGCCTACGGGNG GCWGCAG 3'; R: 5' GTCTCGTGGGCTCGGAGATGTGTATAAGAGACAGGACTACHVGGGTATCTAATCC 3'). The resulting library was sequenced with an Illumina MiSeq instrument using the 2 × 300 bp paired-ends protocol following the manufacturer’s instructions.

To process the paired-end FASTQ files, an amplicon sequence variant (ASV) table was obtained using the DADA2 pipeline (v1.8) in R^45^ following Rosier et al.,^39^. In summary, the forward and reverse reads were trimmed, removing the primer sequences and low-quality bases at the end of the reads through end-trimming. Reads with any ambiguous N base or exceeding 5 expected errors were also discarded. The forward and reverse pairs were merged, with a minimum overlap of 12 bases and a maximum mismatch of 1 base in the overlapping region, to obtain the single denoised variants. After chimera removal, the final amplicon sequence variants (ASVs) were mapped onto the Homo sapiens genome (assembly GRCh38.p13), using Bowtie2^46^ (v2.3.5.1), in order to remove artefactual reads from the host. The Silva database^47,48^ (v138) was set as a reference to assign taxonomy to each ASV. Genus classification was achieved using the DADA2 naive Bayesian classifier method. The ASVs with an assigned genus but without exact species, were aligned using the Blastn tool^49^ (v2.10.0+) against the Silva database with a minimum of 97% of identity.

The absolute and normalized abundance of bacterial genera and species could be computed for graphing and further analysis. Bacterial abundance was normalized using ANCOM-BC approach to account for the compositional nature of 16 S data^50^.

### SMDI score

The Subgingival Microbial Dysbiosis Index (SMDI) was computed for each treatment condition as described by Chen et al.^12^. In short, read counts tables were generated at the species level and normalized by centered log-ratio (CLR) transformation. Based on a list of discriminating species (DS) compiled at different cut-offs of mean decrease in Gini (MDG), which is a measure of the importance of a taxon to the classification, and their mean CLR abundances in health and periodontitis as determined in the study by Chen et al.^12^, a SMDI score was calculated for each inoculum or biofilm sample as follows: SMDI = mean CLR abundance of dysbiotic DS—mean CLR abundance of “normobiotic” (i.e., the composition in health) DS.

### 10-species biofilm model and nitrate

To test the effect of nitrate on oral bacteria, a multi-species in vitro biofilm model containing a synthetic community of 10 oral strains (*Streptococcus mitis* NCTC 12261, *S. intermedius* DSM 20753, *S. oralis* NTCC 11427, *Fusobacterium nucleatum* ATCC 10596, *F. nucleatum spp. vincentii* DSM 19507, *Actinomyces naeslundii* DSM 17233, *Veillonella dispar* NCTC 11831, *Porphyromonas gingivalis* W83, *Prevotella intermedia* DSM 20706, and *Aggregatibacter actinomycetemcomitans*) was used as described by Brown et al. with a nitrate condition. In short, the in vitro biofilms were grown with or without 6.5 mM nitrate for 7 days^23^. A qPCR was performed to quantify the levels of the 10 bacterial species using previously published primer sets^23^. These primers were used to detect *Streptococcus* spp. (F: 5' GATACATAGCCGACCTGAG 3'; R: 5' TCCATTGCCGAAGATTCC 3'), *A. naeslundii* (F: 5' GGCTGCGATACCGTGAGG 3'; R: 5' TCTGCGATTACTAGCGACTCC 3'), *V. dispar* (F: 5' CCGTGATGGGATGGAAACTGC 3'; R: 5' CCTTCGCCACTGGTGTTCTTC 3'), *Fusobacterium* spp. (F: 5' GGATTTATTGGGCGTAAAGC 3'; R: 5' GGCATTCCTACAAATATCTACGAA 3'), *P. gingivalis* (F: 5' GCGCTCAACGTTCAGCC 3'; R: 5' CACGAATTCCGCCTGC 3'), *P. intermedia* (F: 5' CGGTCTGTTAAGCGTGTTGTG 3'; R: 5' CACCATGAATTCCGCATACG 3'), and *A. actinomycetemcomitans* (F: 5’ GAACCTTACCTACTCTTGACATCCGAA 3’; R: 5' TGCAGCACCTGTCTCAAAGC 3').

### Statistical analysis

Statistical analysis of real-time in vitro biofilm growth of periodontal plaque, determination of pH, nitrate, and nitrite in the supernatants, SMDI score and Shannon diversity of the biofilm composition, and qPCR quantification of the 10-species biofilm model was performed using a nonparametric Wilcoxon matched-pairs signed rank test with Bonferroni multiple testing correction for multiple comparisons using IBM SPSS statistics (version 27), and considered statistically significant at a two-sided *p*-value < 0.05. The qPCR data of the 10-species biofilm model was analyzed using a Mann–Whitney *U*-test with Bonferroni–Dunn multiple testing correction.

Fold changes in microbiome composition were tested using ANCOM-BC, and considered statistically significant at an adjusted *p*-value < 0.05^50^. Boruta feature selection^51^ of species identified as significantly different between treatment conditions by ANCOM-BC was applied to further highlight relevant changes. In this study only genera with a median abundance >0.1% were discussed. Data were visualized using GraphPad Prism 8 (version 8.3.0). Other statistical analyses of the bacterial microbiome data were performed according to Johnston et al. using R^9^. Shannon diversity, Adonis tests (Permutational Multivariate Analysis of Variance Using Distance Matrices) and visualization of bacterial composition in a two-dimensional map using constrained correspondence analysis (CCA) were performed using the R Vegan library^52^.

### Reporting summary

Further information on research design is available in the Nature Research Reporting Summary linked to this article.

## Supplementary information

Supplementary information

Reporting Summary

## Data Availability

Sequencing data is available in the NCBI sequence read archive (SRA) under BioProject PRJNA951622. All other data generated or analyzed during this study are included in this published article and its Supplementary Information file or are available from the corresponding author upon reasonable request.
