# Supplementary material for: Nitrate and a nitrate-reducing Rothia aeria strain as potential prebiotic or synbiotic treatments for periodontitis
Source: NPJ Biofilms Microbiomes. 2023 Jun 17;9:40. doi: 10.1038/s41522-023-00406-3 (PMC10276839; doi:10.1038/s41522-023-00406-3)
Supplement: Supplementary file 1 — Supplementary information [file 41522_2023_406_MOESM1_ESM.pdf]

Nitrate and a nitrate-reducing *Rothia aeria* strain as potential prebiotic or synbiotic treatments for periodontitis

Danuta Mazurel[1,2], Miguel Carda-Diéguez[1], Thomas Langenburg[1], Miglė Žiemytė[1], William Johnston[3], Carlos Palazón Martínez[1], Fernando Albalat[4], Carmen Llena[5], Nezar Al-Hebshi[6], Shauna Culshaw[7], Alex Mira[1]†, Bob T. Rosier[1]†

Affiliations:

[1] Genomics & Health Department, FISABIO Institute, Valencia (Spain)

[2] Department of Preventive Dentistry, Academic Center for Dentistry Amsterdam (ACTA), University of Amsterdam and VU University Amsterdam (Netherlands)

[3] Department of Biological and Biomedical Sciences, Glasgow Caledonian University, Glasgow (UK)

[4] Centro Periodontal de Valencia (Spain)

[5] Department of Stomatology, University of Valencia (Spain)

[6] Oral Microbiome Research Laboratory, Kornberg School of Dentistry, Temple University, Philadelphia, Pennsylvania (United States).

[7] Oral Sciences, Glasgow Dental Hospital and School, School of Medicine, Dentistry and Nursing, College of Medical, Veterinary and Life Sciences, University of Glasgow, Glasgow (UK)

†Corresponding authors:

Alex Mira, Department of Health and Genomics, FISABIO Foundation, 46020 Valencia (Spain); tel. +34 961925925, e-mail: mira_ale@gva.es

Bob T. Rosier, Department of Health and Genomics, FISABIO Foundation, 46020 Valencia (Spain); tel. +34 961925967, e-mail: rosier_bob@gva.es

Supplementary Information

Supplemental Table 1. General characteristics and clinical information of patients.

Treatment group	Treatment conditions	Gender (n)		Age (years)	Smoking (n)		Deepest pocket depth (mm)
		M	F	Mean ± SD	Yes	No	Mean ± SD
5 mM nitrate (n = 20)	Control and 5 mM nitrate (n = 9)	3	6	59 ± 9	2	7	7.3 ± 2.0
	Control, 5 mM nitrate, 5 mM nitrate + Ra9, Ra9 (n = 11)	5	5	37 ± 9	5	6	5.7 ± 1.9
	Combined (n = 20)	8	11	45 ± 14	7	13	6.4 ± 2.1
50 mM nitrate (n = 10)	Control and 50 mM nitrate (n = 10)	8	3	52 ± 15	4	6	6.4 ± 1.6
**Total (n = 30)**		16	14	49 ± 14	18	12	6.4 ± 1.9

Ra9: *Rothia aeria* CECT9999.

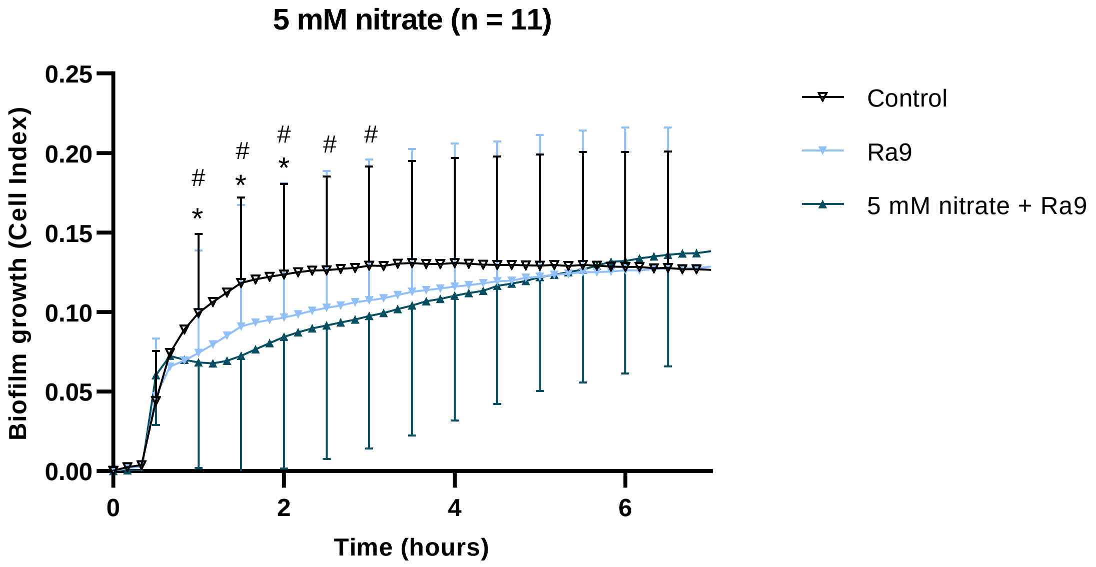

Supplemental Figure 1. Effect of nitrate and *Rothia aeria* supplementation on periodontal biofilm formation. Real-time 7 h growth curve of periodontal plaque biofilms (n = 11) grown *in vitro* supplemented with or without 5 mM nitrate and Ra9. Biofilm growth is expressed as Cell Index (CI) as determined using impedance measurements. Standard deviations are shown at 30 min intervals. Significant differences (p-value < 0.05 according to Wilcoxon matched-pairs signed-rank test) are marked by an asterisk (*) for control versus Ra9 or a number sign (#) for control versus 5 mM nitrate + Ra9. Ra9: *Rothia aeria* CECT9999. The error bars represent the standard deviation of the mean.

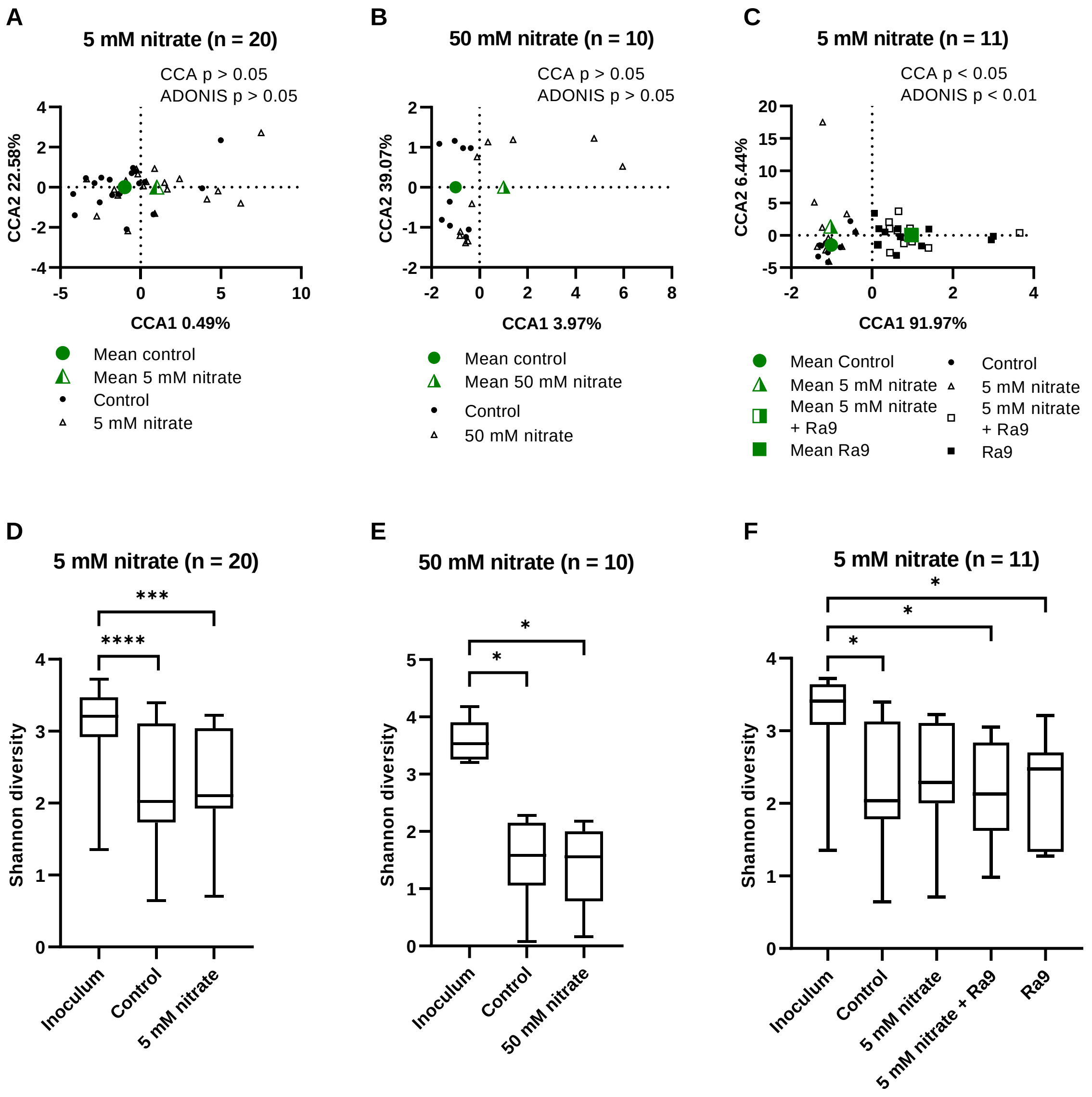

Supplemental Figure 2. Effect of nitrate and *Rothia aeria* supplementation on in vitro periodontal biofilm composition and diversity. CCA-plot of biofilm microbial composition of (**A**) biofilms supplemented with or without 5 mM nitrate (n = 20), (**B**) with or without 50 mM nitrate (n = 10), or (**C**) biofilms supplemented with or without 5mM nitrate or 5mM nitrate and Ra9 (n = 11). Shannon diversity of (**D**) biofilms supplemented with or without 5 mM nitrate (n = 20), and (**E**) with or without 50 mM nitrate (n = 10), or (**F**) biofilms (n = 11) supplemented with or without 5mM nitrate or 5mM nitrate and Ra9. The box and whisker plots show the median (central line), 25th to 75th percentile (box) and min to max (whiskers). Significant differences (adjusted p-value < 0.05

according to Wilcoxon matched-pairs signed rank test with Bonferroni correction for multiple comparisons) are marked by an asterisk (*). Ra9: *Rothia aeria* CECT9999.
