# Supplementary material for: Nitrate and a nitrate-reducing Rothia aeria strain as potential prebiotic or synbiotic treatments for periodontitis
Source: NPJ Biofilms Microbiomes. 2023 Jun 17;9:40. doi: 10.1038/s41522-023-00406-3 (PMC10276839; doi:10.1038/s41522-023-00406-3)
Supplement: Supplementary file 2 — Reporting Summary [file 41522_2023_406_MOESM2_ESM.pdf]

nature portfolio

Corresponding author(s): Bob Rosier
Alex Mira

Last updated by author(s): Feb 7, 2023

Reporting Summary

Nature Portfolio wishes to improve the reproducibility of the work that we publish. This form provides structure for consistency and transparency in reporting. For further information on Nature Portfolio policies, see our Editorial Policies and the Editorial Policy Checklist.

Statistics

For all statistical analyses, confirm that the following items are present in the figure legend, table legend, main text, or Methods section.

n/a	Confirmed	
☐	☒	The exact sample size (n) for each experimental group/condition, given as a discrete number and unit of measurement
☒	☐	A statement on whether measurements were taken from distinct samples or whether the same sample was measured repeatedly
☐	☒	The statistical test(s) used AND whether they are one- or two-sided<br>*Only common tests should be described solely by name; describe more complex techniques in the Methods section.*
☒	☐	A description of all covariates tested
☐	☒	A description of any assumptions or corrections, such as tests of normality and adjustment for multiple comparisons
☐	☒	A full description of the statistical parameters including central tendency (e.g. means) or other basic estimates (e.g. regression coefficient) AND variation (e.g. standard deviation) or associated estimates of uncertainty (e.g. confidence intervals)
☒	☐	For null hypothesis testing, the test statistic (e.g. F, t, r) with confidence intervals, effect sizes, degrees of freedom and P value noted<br>*Give P values as exact values whenever suitable.*
☐	☒	For Bayesian analysis, information on the choice of priors and Markov chain Monte Carlo settings
☒	☐	For hierarchical and complex designs, identification of the appropriate level for tests and full reporting of outcomes
☒	☐	Estimates of effect sizes (e.g. Cohen's d, Pearson's r), indicating how they were calculated

Our web collection on statistics for biologists contains articles on many of the points above.

Software and code

Policy information about availability of computer code

Data collection	No software used, or manufacturer information is provided in the material and methods section.
Data analysis	DADA2 pipeline (v1.8) in R software: create ASV table from FASTQ files with 16s rRNA gene sequencing results.<br>Bowtie 33 (v2.3.5.1) in R: map ASV to human genome.<br>Blastn tool (v2.10.0+) in R: align ASVs with assigned genus but missing species.<br>Graphpad Prism 8 (version 8.3.0): data visualization and statistics<br>R Vegan library: mapping bacterial composition

For manuscripts utilizing custom algorithms or software that are central to the research but not yet described in published literature, software must be made available to editors and reviewers. We strongly encourage code deposition in a community repository (e.g. GitHub). See the Nature Portfolio guidelines for submitting code & software for further information.

Data

Policy information about availability of data

All manuscripts must include a data availability statement. This statement should provide the following information, where applicable:

- Accession codes, unique identifiers, or web links for publicly available datasets
- A description of any restrictions on data availability
- For clinical datasets or third party data, please ensure that the statement adheres to our policy

The sequencing data (FASTQs) will be deposited into a public repository and the accession codes will be available before publication. All other data generated or analysed during this study are included in this published article and its supplementary information files, or are available from the corresponding author upon reasonable request.

Human research participants

Policy information about studies involving human research participants and Sex and Gender in Research.

Reporting on sex and gender	The exact sex and gender information is not relevant for the in vitro experiments presented in this article.
Population characteristics	The participants fulfilled the inclusion and exclusion criteria. Samples taken from participants were subjected to all experimental conditions studied at the time of inclusion and analyzed as paired samples for statistical testing where relevant, thus each participant is represented in each experimental group.
Recruitment	Participants were recruited after clinical assessment of eligibility and periodontitis diagnosis. Participation was voluntary and participation did not affect the patient or their treatment. Samples taken from participants were subjected to all experimental conditions studied at the time of inclusion (no allocation bias).
Ethics oversight	The study protocol was reviewed and approved by the Ethics Committee of the University of Valencia (Spain).

Note that full information on the approval of the study protocol must also be provided in the manuscript.

Field-specific reporting

Please select the one below that is the best fit for your research. If you are not sure, read the appropriate sections before making your selection.

☒ Life sciences ☐ Behavioural & social sciences ☐ Ecological, evolutionary & environmental sciences

For a reference copy of the document with all sections, see nature.com/documents/nr-reporting-summary-flat.pdf

Life sciences study design

All studies must disclose on these points even when the disclosure is negative.

Sample size	No sample-size calculation has been performed. Sample size is based on previous work with complex oral samples of healthy individuals and nitrate (see References 13 and 20) and the maximum number of participants recruited during the duration of the study.
Data exclusions	No sample or data was excluded from the study.
Replication	Samples taken from participants were subjected to all experimental conditions studied at the time of inclusion and analyzed as paired samples for statistical testing where relevant, thus each participant is represented in each experimental group, and consistent changes are detected. Findings were reproducible over different experimental groups receiving different experimental conditions, and in line with previous research from the authors of the study in healthy individuals. Effects observed in the samples of clinical origin corresponded to those found in an vitro synthetic community by a different research group as included in this study.
Randomization	All clinical samples were subjected to each experimental condition studied at the moment of inclusion, therefore randomization took place
Blinding	Not applicable. All clinical samples were subjected to all experimental condition studied at the moment of inclusion and analyzed as paired samples for statistical testing where relevant. Processing and handling was the same for all samples and appropriate controls were included.

Reporting for specific materials, systems and methods

We require information from authors about some types of materials, experimental systems and methods used in many studies. Here, indicate whether each material, system or method listed is relevant to your study. If you are not sure if a list item applies to your research, read the appropriate section before selecting a response.

Materials & experimental systems

n/a	Involved in the study
☒	☐ Antibodies
☒	☐ Eukaryotic cell lines
☒	☐ Palaeontology and archaeology
☒	☐ Animals and other organisms
☒	☐ Clinical data
☒	☐ Dual use research of concern

Methods

n/a	Involved in the study
☒	☐ ChIP-seq
☒	☐ Flow cytometry
☒	☐ MRI-based neuroimaging
